# Matlab Open Source Code: Noise-Assisted Multivariate Empirical Mode Decomposition Based Causal Decomposition for Causality Inference of Bivariate Time Series

**DOI:** 10.3389/fninf.2022.851645

**Published:** 2022-06-16

**Authors:** Yi Zhang, Guan Wang, Ziwen Li, Mingjun Xie, Branko Celler, Steven Su, Peng Xu, Dezhong Yao

**Affiliations:** ^1^School of Aeronautics and Astronautics, University of Electronic Science and Technology of China, Chengdu, China; ^2^Key Laboratory for Neuro Information of Ministry of Education, School of Life Science and Technology, University of Electronic Science and Technology of China, Chengdu, China; ^3^Center for Information in BioMedicine, University of Electronic Science and Technology of China, Chengdu, China; ^4^Glasgow College, University of Electronic Science and Technology of China, Chengdu, China; ^5^Biomedical Systems Laboratory, University of New South Wales, Sydney, NSW, Australia; ^6^Center for Health Technologies, Faculty of Engineering and Information Technology, University of Technology, Sydney, NSW, Australia

**Keywords:** NA-MEMD Causal Decomposition, empirical mode decomposition, Granger causality, CCM, Causal Decomposition, causality inference, bivariate time series

## Abstract

Causality inference has arrested much attention in academic studies. Currently, multiple methods such as Granger causality, Convergent Cross Mapping (CCM), and Noise-assisted Multivariate Empirical Mode Decomposition (NA-MEMD) are introduced to solve the problem. Motivated by the researchers who uploaded the open-source code for causality inference, we hereby present the Matlab code of NA-MEMD Causal Decomposition to help users implement the algorithm in multiple scenarios. The code is developed on Matlab2020 and is mainly divided into three subfunctions: *na*_*memd*, *Plseries*, and *cd*_*na*_*memd*. *na*_*memd* is called in the main function to generate the matrix of Intrinsic Mode Functions (IMFs) and *Plseries* can display the average frequency and phase difference of IMFs of the same order in a matrix which can be used for the selection of the main Intrinsic Causal Component (ICC) and ICCs set. *cd*_*na*_*memd* is called to perform causal redecomposition after removing the main ICC from the original time series and output the result of NA-MEMD Causal Decomposition. The performance of the code is evaluated from the perspective of executing time, robustness, and validity. With the data amount enlarging, the executing time increases linearly with it and the value of causal strength oscillates in an ideally small interval which represents the relatively high robustness of the code. The validity is verified based on the open-access predator-prey data (wolf-moose bivariate time series from Isle Royale National Park in Michigan, USA) and our result is aligned with that of Causal Decomposition.

## 1. Introduction

In early studies, the definition of Noise-assisted Multivariate Empirical Mode Decomposition (NA-MEMD) based Causal Decomposition was given by Ur Rehman and Mandic (2011), She et al. (2017), and Zhang et al. (2017, 2021), dealing with the cause-effect relationship observed across from two time series signals in the real-world situations (Adarsh and Janga Reddy, 2019). The study provides Matlab Open Source Code of NA-MEMD Causal Decomposition and helps scholars and researchers (particularly in function connectivity Mueller et al., 2013; Meshi et al., 2016, signal detection and processing Abbate et al., 1997; Song and Que, 2006, and Statistical Causality Cox, 1992; Cox and Wermuth, 2004) to determine the causality occurring at stochastic, deterministic and complex dynamical (nonlinear deterministic) processes on the basis of time series (Small, 2008). The development of such a theoretical framework has been arising since the publication of Granger causality (Granger, 1969; Kamiński et al., 2001; Seth, 2007). It defines that variable X is considered to be the Granger cause of variable Y if X helps to explain future changes in Y. The test of Granger causality introduces F-test to quantify the autoregressive property between X and Y by solving a best least-square problem. Granger causality was fundamentally based on the hypothesis of uncoupling cause-effect and, thus, would be applicable to stochastic processes. For multivariate time series, Looney et al. (2018) propose a novel multivariate sample entropy that can handle the analysis of within- and cross-channel dynamics. It is also the only method to identify synchronized regularity dynamics. In addition, inspired by Takens' Embedding Theorem (Noakes, 1991), Sugihara et al. proposed Convergent Cross Mapping (CCM) (Sugihara et al., 2012; Krakovská et al., 2015), which held that if the projections of X and Y in a certain dimension, i.e., X′ and Y′ respectively, existed the causal relationship, then X and Y would have CCM causality. It was assumed that a cause-effect relation was embedded in a complex dynamical process which was also likely to be a linear/nonlinear deterministic system. CCM accommodated the inseparability/coupling of causal effects. Not surprisingly, Yang et al. (2018) established Causal Decomposition and further confirmed the utility of Hilbert-Huang Transform (HHT) (Huang, 2014a,b) in causality inference. Cause-effect mutual information was assumed to be carried over the instantaneous phase of the observed time series. However, standard methods such as Standard Fourier, wavelet, and Hilbert may encounter problems in analyzing real-world data. First, the approaches depending on predefined function heavily depend on the data length and stationarity and the real-world data which are often short and intermittent may hinder the analysis process. Second, among the standard methods, integral transforms pursue more frequency concepts than the temporal concept which may increase the frequency resolution but lose information in the time domain. Third, for nonmonocomponent data, the direct implementation of analytic signal representation results in negative IFs which have no physical justification. Next, accurate observation and measurement should be conducted on the corresponding scales rather than globally. However, correlation, coherence, and synchrony measure cannot be performed on certain scales (Mandic et al., 2013). Causal Decomposition was based on phase dependency, which was distinguished from the prediction paradigm such as Granger causality, CCM, Mutual Information from Mixed Embedding (Kugiumtzis, 2013; Jia et al., 2019), Dynamic Causal Modeling (Friston et al., 2003, 2013), and Transfer Entropy (Staniek and Lehnertz, 2008; Bossomaier et al., 2016). It was suitable for complex dynamical processes, acquired in the manner of time series in certain similar time scales. The development of the MEMD theoretical framework has been started by Altaf et al. (2007) who put forward a method that develops a mathematical approach to adapt EMD to both real and complex domains. The so-generated Intrinsic Mode Functions (IMFs) can be used in processing both real and complex signals. Another method proposed by Tanaka and Mandic (2007) also provides an insight into extending standard EMD to the complex domain. It takes advantage of both positive and negative frequency components of signals to generate complex-valued IMFs. Since the EMD is initially limited to real-valued time series, an extension of the EMD framework to bivariate time series is designed by extracting zero-mean rotating components (Rilling et al., 2007). In order to make EMD compatible with trivariate signals, ur Rehman and Mandic (2009) comes out with a theoretical framework that projects local mean envelop to multiple directions in three-dimensional spaces adapting the rotation property of quaternions. In order to handle the causality analysis in multiscale time series, like multiple physiological time series (e.g., EEG, EMG, and ECG) composed network (Bashan et al., 2012; Faes et al., 2017), we recently presented NA-MEMD Causal Decomposition (Zhang et al., 2021) and pointed out its potential to the causality inference in a complex dynamical process. In Zhang et al. (2017), multichannel EMG signals are processed by EEMD, MEMD, and NA-MEMD, and their outcomes are quantitively assessed by comparing their number of IMFs, mode-alignment, and mode-mixing. It has been justified that NA-MEMD has a relatively outstanding performance in processing brain-muscle signals compared with EEMD and MEMD. Many studies have contributed to algorithm implementations related to causality analyzes in open-source scenarios. C and C++ code of EMD using Matlab Coder™ was introduced in R2018a in MathWorks (Huang, 2022). Furthermore, Rehman and Mandic generated the algorithm of Multivariate empirical mode decomposition (MEMD) (Rehman and Mandic, 2010). Then, Zhang et al. (2017) published a Matlab toolbox (Wen et al., 2022) of NA-MEMD in 2017. In the causality analysis, Mønster provided a Convergent Cross Mapping algorithm in MATLAB in 2018 (Jakubik, 2022). Yang published the proposed Causal Decomposition approach in GitHub (Yang, 2022), which then was exchanged to MathWorks in 2020. In the study, we hereby present the Matlab code package for NA-MEMD Causal Decomposition used in the preliminary article by Zhang et al. (2021), offering the configuration specification details required in the data analyzes and tests, and providing the functional specification of line-by-line codes in the workflow.

## 2. Specification of Calling Function

### 2.1. Background Theory

**Figure 1:**
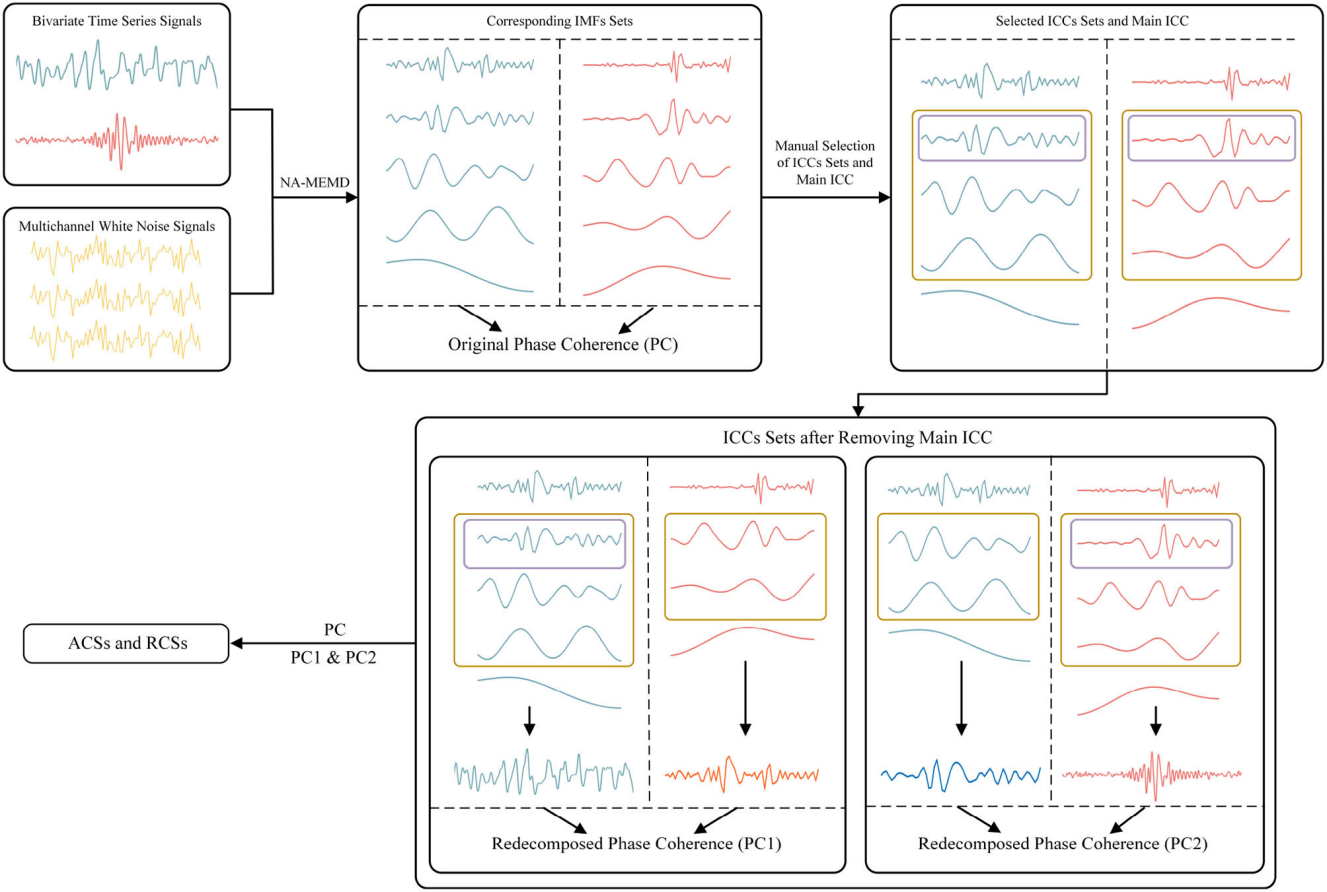
Basic workflow for NA-MEMD Causal Decomposition.

The overall procedure for NA-MEMD Causal Decomposition is illustrated in Figure 1 by 5 steps. It first adds multichannel auxiliary white noise (shown in step **a-2**) to original bivariate time series signals (shown in step **a-1**), followed by NA-MEMD to obtain two corresponding IMFs sets (shown in steps **b-1** and **b-2**) as well as their phase coherence. Next, the main ICC (framed by the purple rectangles in steps **c** and **d**) and ICCs sets (framed by the yellow rectangles in steps **c** and **d**) are selected according to average frequency and phase difference. Only the IMFs with identical frequency scales and small phase differences can be regarded as ICCs. The main ICC is usually selected as the first ICC in the set. Removing the main ICC from IMFs sets and adding the remaining signals in each IMFs setup, two pairs of signals are formed by one modified signal without the main ICC and the other original one (shown in step **d**). The phase coherence of the two pairs is calculated. Combined with the value of the phase coherences of two redecomposed signal pairs and the original signal pair, the value of Absolute Causal Strengths (ACSs) and Relative Causal Strengths (RCSs) can be obtained by NA-MEMD Causal Decomposition (shown in step **e**).

### 2.2. Function *na*_*memd*

**Figure 2:**
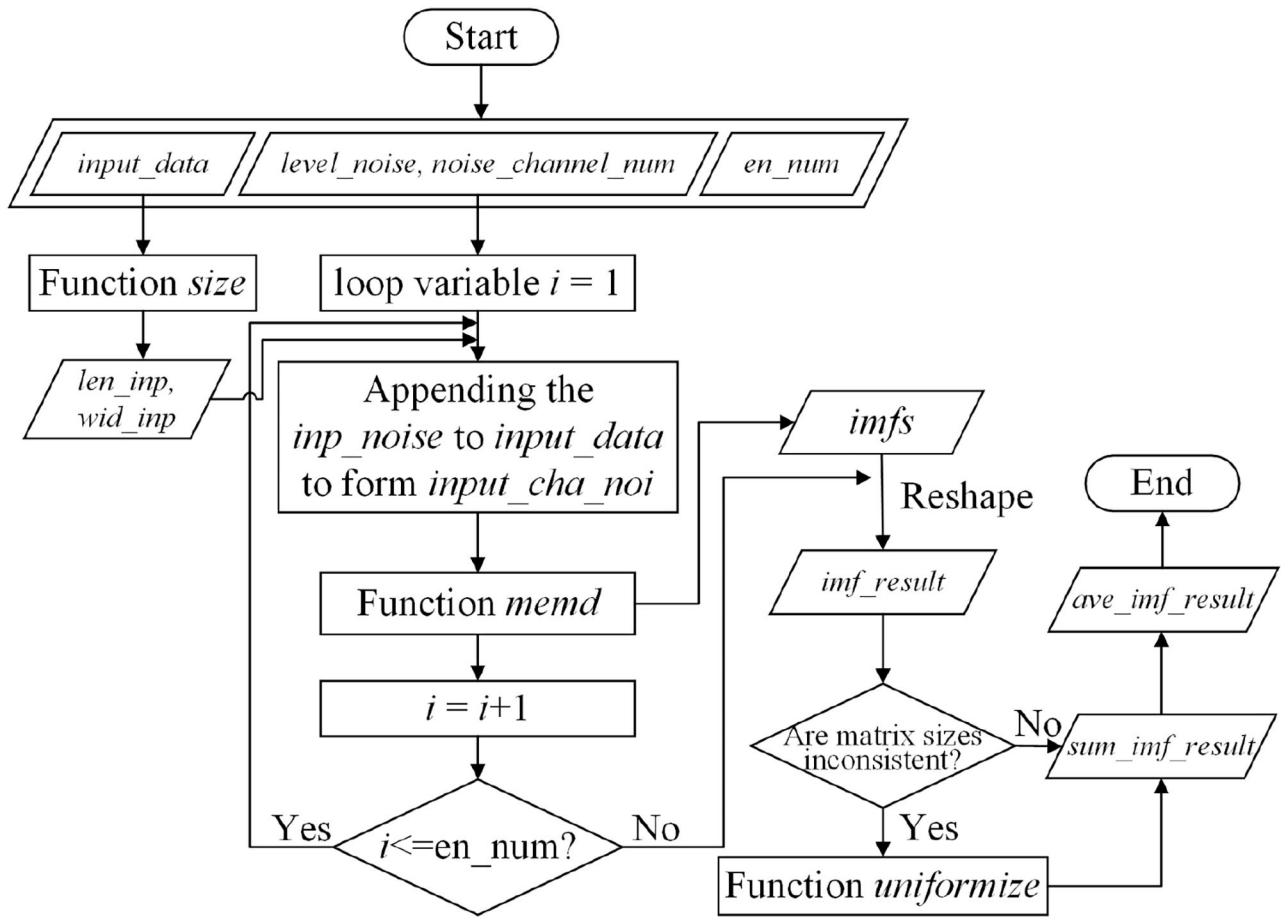
The workflow of function *na*_*memd*.

The procedure for function *na*_*memd* is illustrated in Figure 2. The function enables to form a matrix, giving Intrinsic Mode Functions (IMFs) decomposed from NA-MEMD with causal-effect time series defined by input matrix *input*_*data*. Relevant variables about function *na*_*memd* are listed in Table 1. The function *na*_*memd* is invoked when variables *input*_*data*, *ave*_*noise*, *level*_*noise*, *noise*_*channel*_*num*, and *en*_*num* are set. Then, function *size* returns the number of row elements of matrix *input*_*data* to variable *len*_*inp*, and the number of column elements of matrix *input*_*data* to variable *wid*_*inp*. *level*_*noise* and *noise*_*channel*_*num* are used to generate the random Gaussian-White noise time series. After then, function *memd* is run if vector/matrix *inp*_*noise* is appended to matrix *input*_*data*, which is named matrix *input*_*cha*_*noi*. It repeats for constant *en*_*num* times (*i* is the loop variable). For each repetition, a three-dimension array *imfs* is returned which contains all IMFs decomposed from matrix *input*_*cha*_*noi*. In order to facilitate the ensemble process of NA-MEMD, array *imfs* is reshaped into cell *imf*_*result*. Considering repetitions in-between, if the matrix sizes in cell *imf*_*result* perhaps are inconsistent, the residual is embedded with a pseudo-monotone variation. In order to guarantee the consistency of the size in cell *imf*_*result*, the residuals can be ignored together. Matrix elements of *imf*_*result*, *sum*_*imf*_*result*, and *imf*_*result* are first initialized to be zero, which stand for reshaped IMF data, the summation of the reshaped IMF, and the average value of the summation respectively. The noise data are generated by random alternating noise components determined by parameter *level*_*noise*. This process is repeated by times of *en*_*num*. For each loop, the noise is appended to matrix *input*_*data* which is matrix *input*_*cha*_*noi*. Then matrix *imfs* is calculated by performing MEMD on *input*_*cha*_*noi* with function *memd*. Next, *imfs* is reshaped into *imf*_*result* for further summation which data is stored in *sum*_*imf*_*result*. The average value is also calculated and assigned to *ave*_*imf*_*result*.

**Table 1:** Parameter configurations for function ***na*_*memd***.

**Parameter names**	**Parameter specification**	**Configurations**
*input*_*data*	Data type:***Matrix*** IMFs decomposed from NA-MEMD	Two time series data on identical length.
*level*_*noise*	Data type: ***Number*** Used to determine the intensity of alternating component of noise in *na*_*memd*	With it increasing, the represented cause-effect relationship may be attenuated.
*noise*_*channel*_*num*	Data type: ***Number*** Used to determine the channel number of appended noise signals in *na*_*memd*	With it increasing, the represented cause-effect relationship may be attenuated.
*en*_*num*	Data type: ***Number*** Used to determine the cycle index of noise-assisted calculation in *na*_*memd*	With it increasing, the running time will be prolonged dramatically.

### 2.3. Function *PLseries*

**Figure 3:**
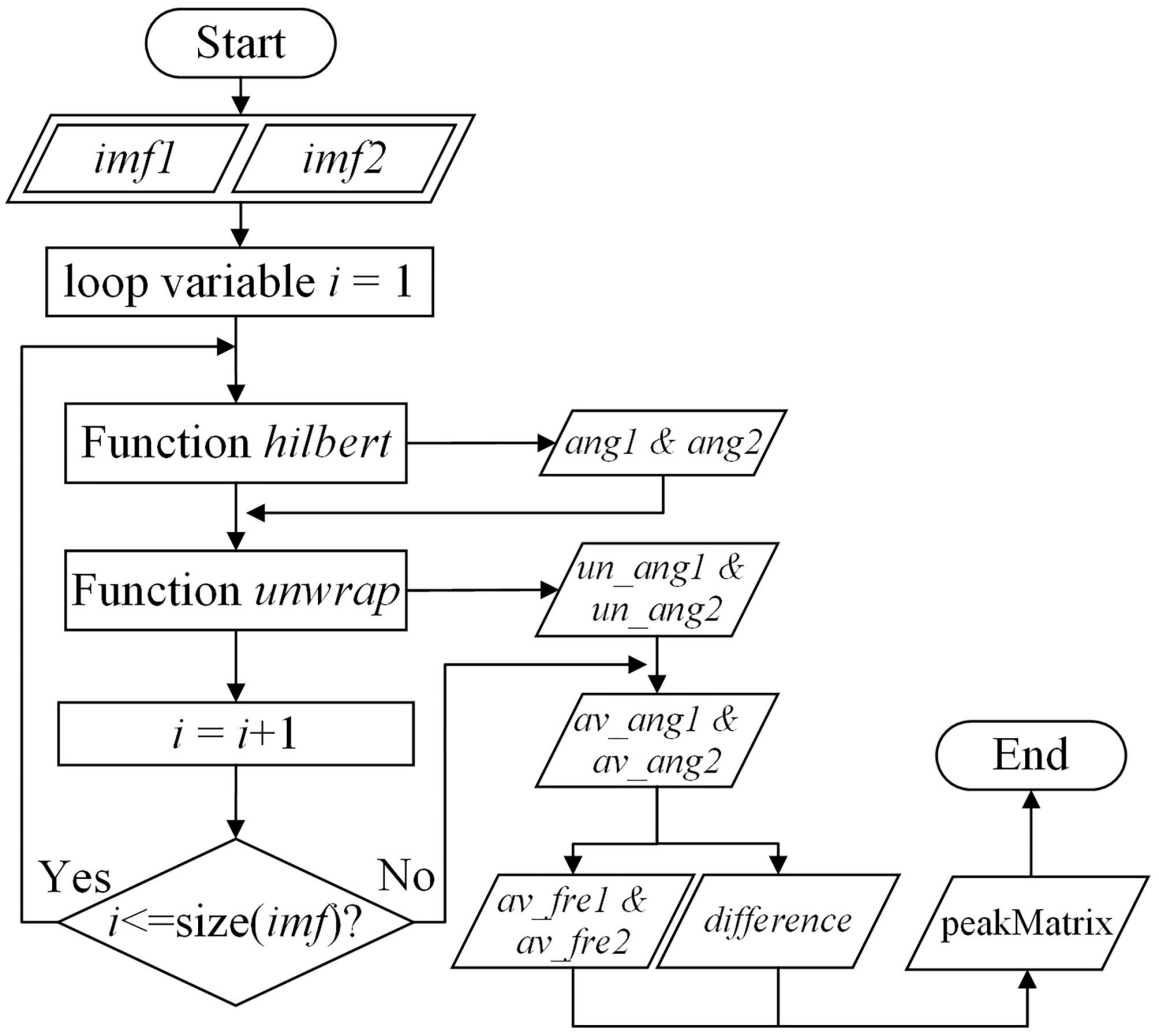
The workflow of function *PLseries*.

The workflow for function PLseires is shown in Figure 3. The function outputs a matrix that shows the average frequency and their phase difference of IMFs decomposed from NA-MEMD by matrices *imf*1 and *imf*2 (Table 2), respectively. The row of *imf*1 and *imf*2 stand for the index of IMFs and the column of *imf*1 and *imf*2 refers to the length of the time series. The output matrix is defined as matrix *peakMatrix*. The function PLseries is called when matrix *imf*1 and *imf*2 are input. The elements in vectors *av*_*fre*1, *av*_*fre*2, and *difference* are initialized to be zero and their length of the row is also set to be consistent with that of *imf*1 and *imf*2 (their rows are in the same length). After that, for each IMF, Hilbert Transform is used to calculate its instantaneous phase. To alleviate the effect of phase winding, the function *unwarp* is called to obtain the real phase which is defined as vectors *un*_*ang*1 and *un*_*ang*2. Then the average value of the unwrapped phase is calculated and stored in vectors *av*_*ang*1 and *av*_*ang*2, respectively. Their difference is stored in vector *difference*. According to vectors *un*_*ang*1 and *un*_*ang*2, the phase information is converted to frequency information which is stored in vectors *fre*1 and *fre*2, respectively. Their average values are calculated and are stored in vectors *av*_*fre*1 and *av*_*fre*2. The final output matrix *peakMatrix* is the combination of vectors *av*_*fre*1, *av*_*fre*2, and *difference* which are in the first, the second, and the third column, respectively.

**Table 2:** Parameter configurations for function ***PLseries***.

**Parameter names**	**Parameter specification**	**Configurations**
*imf*_1, *imf*_2	Data type:***Matrix*** IMFs decomposed from NA-MEMD	Two time series data on identical length.
*ave*_*fre*1, *ave*_*fre*2	Data type:***Vectors*** Represent the average instantaneous frequency of two IMF sets	Null
*difference*	Data type: ***Vector*** Represent the average phase difference of two IMF sets	Null
*peakMatrix*	Data type: ***Matrix*** A matrix to illustrate *av*_*freq*1,*av*_*freq*2 and *difference* in different columns	The first two columns refer to *av*_*freq*1 and *av*_*freq*2. The third column represents *difference*.

### 2.4. Function *causal*_*decomposition*

**Figure 4:**
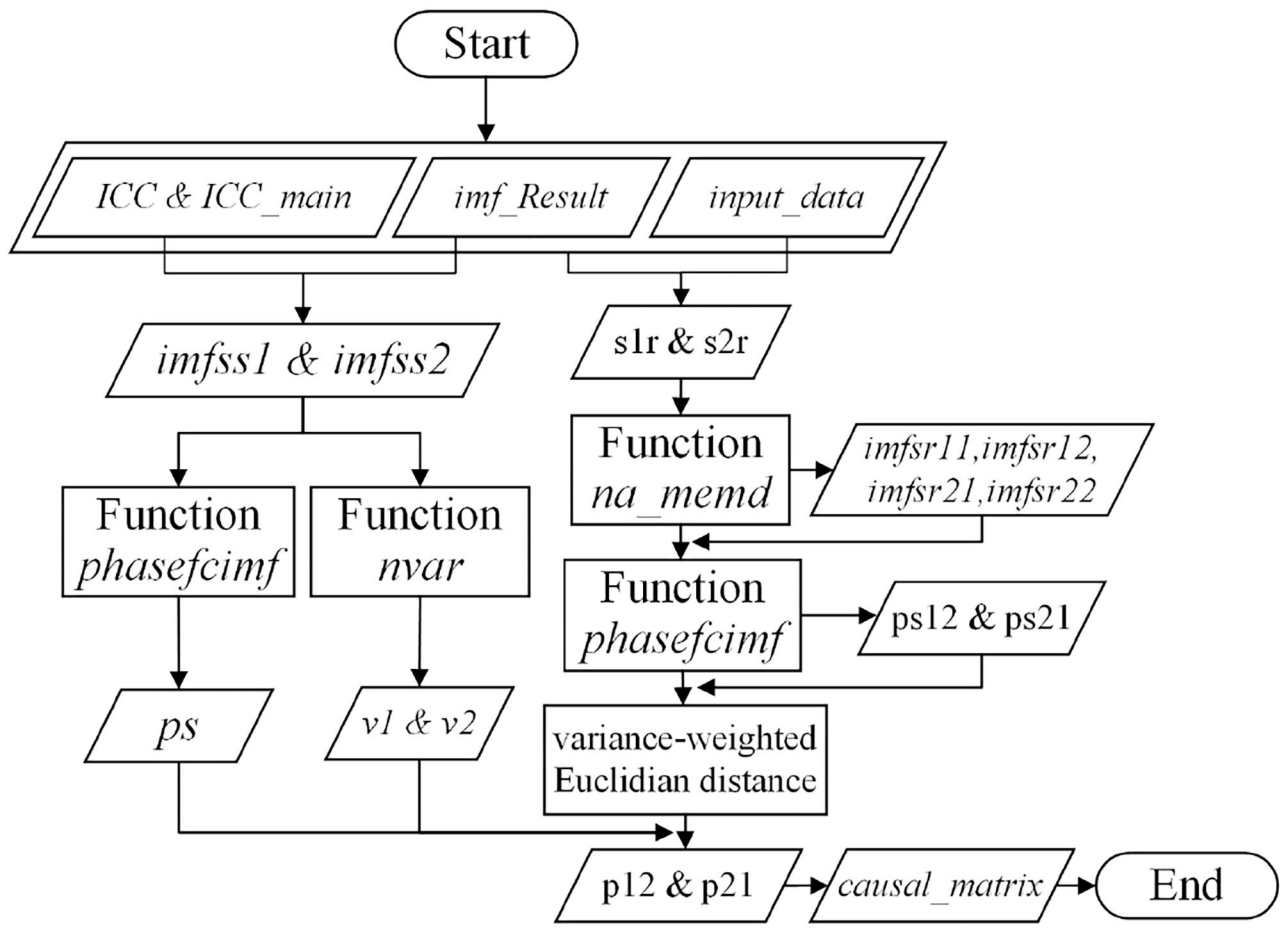
The workflow of function *causal*_*decomposition*.

The workflow for function causal_decomposition is demonstrated in Figure 4. The function generates a matrix *causal*_*matrix* to provide values of RCSs and ACSs according to the times series inputted by matrix *input* referred to in Table 3. The raw data of bivariate time series are row-by-row loaded to input which is subsequently defined as vectors *s*1 and *s*2. Those two decomposed IMF sets are then assigned as matrices *imfs*1 and *imfs*2, respectively. Intrinsic Causal Components (ICCs) sets, vector *ICC*, are manually reviewed and selected by function *plot*_*and*_*pick*. Based on *ICC*, the number *ICC*_*main* is confirmed to indicate RCSs and ACSs of raw bivariate time series of input. Main ICC is selected manually by function *plot*_*and*_*pick*, and is defined as vector *imfss*1 and *imfss*2. Their phase coherence and variance are calculated by function *phasefcimf* and function *nvar*, respectively. After obtaining the main ICC, it is removed from the corresponding inputs *s*1 and *s*2, and the rest are defined as vectors *s*1*r* and *s*2*r*. They are used to replace the corresponding signal in input to form two new input matrices *input*1 and *input*2. Then they are re-decomposed by calling function *na*_*memd* to calculate two IMFs pairs (matrices *imfsr*11, *imfsr*12 and matrices *imfsr*21, *imfsr*22, respectively). The phase coherence between paired IMFs sets and the distance between the phase coherences of the original IMFs pairs and the redecomposed ones are calculated and stored in (*ps*12, *ps*21) and (*p*12, *p*21), respectively. The final causal strengths are outputted by the matrix *causal*_*matrix*.

**Table 3:** Parameter configurations for function ***causal***_***decomposition***.

**Parameter names**	**Parameter specification**	**Configurations**
*ICC*_*main*	Data type:***Number*** The index of selected main ICC	The main ICC is the one with the minimal phase difference.
*input*	Data type:***Matrix*** The raw data to be analyzed	Null
*imf*_*result*	Data type: ***Matrix*** The result from *na*_*memd* in *na*_*memd*_*causal*_*decomposition*	Null
*level*_*noise*	Data type: ***Number*** Used to determine the intensity of alternating component of noise in *na*_*memd*	With it increasing, the represented cause-effect relationship may be attenuated.
*noise*_*channel*_*num*	Data type: ***Number*** Used to determine the channel number of appended noise signals in *na*_*memd*	With it increasing, the represented cause-effect relationship may be attenuated.
*en*_*num*	Data type: ***Number*** Used to determine the cycle index of noise-assisted calculation in *na*_*memd*	With it increasing, the running time will be prolonged dramatically.
*causal*_*matrix*	Data type: ***Matrix*** Used to output the relative causal strengths and the absolute strengths.	The first two columns represent the relative causal strengths. The latter two columns represent the absolute causal strengths.

## 3. Result

### 3.1. Test for Time Series Data

To guarantee the experimental efficiency in the real application, variable ***noise***_***channel***_***num*** and ***en***_***num*** and the data length should be modified. The input data is the Gaussian-White noise with a mean value of 0 and variance of 1, and the experiment is performed on Matlab 2020b on the fully powered laptop.

#### 3.1.1. Variable *noise*_***channel***_***num***

The relationship between ***noise***_***channel***_***num*** and executing time is demonstrated below in Table 4.

**Table 4:** The relationship between *noise*_*channel*_*num* and executing time.

***noise***_***channel***_***num***	**3**	**4**	**5**	**6**	**7**	**8**	**9**	**10**
Executing time(s)	24.50	25.68	26.86	28.01	31.73	33.58	34.80	37.64

In this test, ***en***_***num*** and data length are determined to be 3 and 500, respectively. It is clear that executing time has a linearly increasing trend with *noise*_*channel*_*num* from the recorded data in Table 4.

#### 3.1.2. Variable *en*_*num*

The relationship between ***en***_***num*** and executing time is demonstrated below in Table 5.

**Table 5:** The relationship between *en*_*num* and executing time.

***en***_***num***	**5**	**10**	**15**	**20**	**25**	**30**	**35**	**40**
Executing time(s)	41.26	78.94	117.09	155.09	194.79	217.20	245.20	288.27

In this test, ***noise***_***channel***_***num*** and data length are fixed to be 3 and 500, respectively, and with ***en***_***num*** increasing, executing time increases linearly along with ***en***_***num*** according to Table 5.

#### 3.1.3. Data Length

The relationship between **data length** and executing time is demonstrated below in Table 6.

**Table 6:** The relationship between data length and executing time.

**Data length**	**250**	**500**	**750**	**1,000**	**1,250**	**1,500**	**1,750**	**2,000**
Executing time(s)	16.11	22.46	31.49	37.65	44.14	50.87	62.34	70.41

In this test, ***noise***_***channel***_***num*** and ***en***_***num*** are both fixed to be 3, and with **data length** increasing, executing time increases linearly along with **data length** referred to in the data in Table 6.

### 3.2. Robustness and Validity Test

Since the random noise is involved in NA-MEMD, it is likely that the results of different execution will be slightly different from one another. Therefore, the consistency of the output should be tested. In this test, **level_noise**, **noise_channel_num**, **en_num**, and **data length** are set to be 0.001, 3, 5, and 61, respectively, and the open-access predator-prey data is provided by Vucetich and Peterson (2012). The result is shown in Table 7.

**Table 7:** Result of robustness and validity test.

**Test number**	**RCSs of X-to-Y (*C*_*x*_*y*)**	**RCSs Y-to-X (*C*_*y*_*x*)**	**ACSs of X-to-Y (*C*_*x*_*y*)**	**ACSs Y-to-X (*C*_*y*_*x*)**
1	0.7168	0.2832	0.2237	0.0884
2	0.5374	0.4626	0.1927	0.1659
3	0.6804	0.3196	0.2003	0.0941
4	0.7594	0.2306	0.1810	0.0542
5	0.6364	0.3636	0.2048	0.1170
6	0.6042	0.3958	0.1971	0.1291
7	0.6398	0.3602	0.2038	0.1148
8	0.6585	0.3415	0.1974	0.1024
9	0.5943	0.4057	0.1923	0.1313
10	0.5473	0.4527	0.1979	0.1637

In Table 7, since the X-to-Y causality value and Y-to-X causality value sum to one, only the X-to-Y causal value is considered in the stability discussion.

From the statistical point of view, the mean value of ***C***_***xy***_ is 0.6384 and the variance is 0.004712 which is less volatile. Combined with the actual causal process demonstrated by the causality values by Yang et al. (2018), all of them indicate strong and valid causality since our results are close to that of Yang et al. (2018), which demonstrates the outcome of the procedure is consistent with the algorithm.

## 4. Discussion

### 4.1. The Approach to Exert Noise in Original Signals in *na*_*memd*

Generally, input parameters *level*_*noise*, *en*_*num*, and *noise*_*channel*_*num* are used to determine the total amount of noise appended to the original signal. The description of the relevant variables can be checked in Table 3. Before setting the parameters, the noise level in the original input data should be evaluated. If the input has already been overwhelmed by noise, both the two parameters are supposed to be set higher to better remove the noise in the original data. However, with the Gaussian-White noise increasing, the effective data in the input is also likely to be attenuated by the appended noise. As a result, it is of significance to choose a proper value of *level*_*noise* and *noise*_*channel*_*num* to avoid excessive Gaussian-White noise. One method of removing the original noise without doing damage to the effective data is to reduce the product of *level*_*noise* and *noise*_*channel*_*num* and to increase *en*_*num*.

### 4.2. The Method of Selecting ICCs Set From IMFs

For ICCs, the ICCs set and the main ICC need to be selected for further operation. Among all the IMFs, ICCs are the ones whose average frequencies and phases are on the same scale. IMFs in different frequency scales or with large phase differences should be excluded from the ICCs set. In ICCs set, the main ICC is the one with the smallest average phase difference. Besides, the phase diagram of IMFs generated by Matlab can also be used to discriminate ICCs from all IMFs. Assist from both data in *peakMatrix* and visual aid of signal diagram is necessary for ICCs discrimination.

### 4.3. Calculation of RCSs and ACSs

In this code, it is recommended that only RCSs are used to detect the causal-effect relationship between bivariate time series signals.

Absolute Causal Strengths is defined as the variance weighted Euclidean distance (De Leeuw and Pruzansky, 1978) between the re-decomposed IMFs set and the original IMFs set. Its value can reflect the relative cause-effect relationship between multiple signals. The signals with higher ACS can be regarded as the cause and the ones with smaller ACS can be considered as the effect, respectively. Specifically, if only two signals are considered, RCS can also be calculated to illustrate their cause-effect relationship. It normalizes the ACS into an interval between zero and one, and we use the value of RCS to interfere with the causal-effect relationship. When the value of RCS is larger than 0.5, it represents the relationship of cause, and when the value of RCS is smaller than 0.5, it represents the relationship of effect. When the value is equal to 0.5, the relationship can be either reciprocal causation or an irrelevant relationship. When the ACS of two candidate signals is negligible, RCS will converge to 0.5. The threshold value is normally set as 0.05 (Yang et al., 2018).

### 4.4. Further Analysis of the Result

#### 4.4.1. The Reason for Linear Increase in Executing Time

In this code, the ensemble process is designed as simple loops for multiple noise appending rather than nested loops or complex function iteration. According to ur Rehman et al. (2013), compared with EEMD, NA-MEMD applies an ensemble algorithm that massively reduces the reconstruction error. As a result, the impact of the number of noise channels on creating error can be negligible. However, ur Rehman et al. (2013) recommend the number to be 2 which is suitable for the test situation and can be adjusted accordingly. Therefore, this experiment can change the **noise_channel_num** and observe differences. As a result, the executing time is in direct proportion to the total data size. When **noise_channel_num**, **en_num**, and **data length** increase, respectively, the total data size inflates linearly. Therefore, the executing time would have a tendency of linear increase with those variables.

#### 4.4.2. The Determination for System Robustness

For any existing causality pairs, their cause-effect relationship is stable. RCS between them is considered a constant. As a result, it is appropriate to use the variance of relative causality data to represent the robustness of the system. If the variance has a small value which means RSC is less likely to fluctuate between the ideal causality strength, the system has relatively high robustness. If the variance is measured high, then it proves that the system is more susceptible to the external or internal environment.

## 5. Conclusion

A code package of NA-MEMD has been proposed to facilitate the potential users to apply the algorithm in an effective way. The users need to select the ICCs set and main ICC to check the causal-effect relationship according to the printed frequency-phase matrix and the signal figures. Based on MEMD, the crucial step is to append multi-channel random Gaussian-White noise to the original signal repeatedly to attenuate self-carrying noise. By adjusting the number of noise channels, the intensity of appended noise per channel, and the cycle index, it has been shown that the users can alleviate the influence of original noise without damaging the original data. As a result, NA-MEMD Causal Decomposition has potential for applications in analyzing bivariate and multiscale time series signals.

## 6. Data Availability Statement

The code of NA-MEMD Causal Decomposition for causality inference of bivariate time series will be available through open-source platform github, https://github.com/AaronLi43/ginkgo_glasgow. The demo data used in section RESULT is Wolf and moose field data which are available online at the United States Isle Royale National, https://isleroyalewolf.org/data/data/home.html. Further inquiries can be directed to the corresponding author.

## Author Contributions

YZ, GW, and ZL found the potential utility of the theoretical framework of NA-MEMD Causal Decomposition in function connectivity, signal detection and processing, Statistical Causality, implemented Matlab open-source code for NA-MEMD Causal Decomposition, tested the efficiency, robustness, validity of the code, carried out the results and discussions, and drafted and revised the manuscript. MX participated in Section Result in part. SS verified and proved the theoretical part of NA-MEMD Causal Decomposition. DY and PX supervised the project and checked the paper quality. PX advised the submission procedure and the manuscript quality linked toward its extensions to fMRI studies. All authors contributed to the article and approved the submitted version.

## Funding

This study was supported by the National Natural Science Foundation of China (#61961160705, #U19A2082, #62103085, and #61801094), Sichuan Science and Technology Program (#2021YFG0126), Major Science and Technology Special Projects in Sichuan (#2020YFG0469), and Fundamental Research Funds for the Central Universities China (#ZYGX2019J086).

## Conflict of Interest

The authors declare that the research was conducted in the absence of any commercial or financial relationships that could be construed as a potential conflict of interest.

## Publisher's Note

All claims expressed in this article are solely those of the authors and do not necessarily represent those of their affiliated organizations, or those of the publisher, the editors and the reviewers. Any product that may be evaluated in this article, or claim that may be made by its manufacturer, is not guaranteed or endorsed by the publisher.
